# Road to zero: Research and industry perspectives on zero-emission commercial vehicles

**DOI:** 10.1016/j.isci.2023.106751

**Published:** 2023-04-26

**Authors:** Matteo Muratori, Brennan Borlaug, Catherine Ledna, Paige Jadun, Aravind Kailas

**Affiliations:** 1 National Renewable Energy Laboratory, Golden, CO 80401, USA; 2 Volvo Group North America, Costa Mesa, CA 92626, USA

**Keywords:** Energy transportation, Civil engineering, Transportation engineering, Energy Systems

## Abstract

Medium-and heavy-duty vehicles (MHDVs) comprise only a small fraction of vehicles on the road but disproportionately contribute to greenhouse gas emissions and air pollution from the transportation sector. Given the large variety of vehicle types—ranging from heavy-duty pickup trucks and box trucks to full-size buses and Class 8 tractor semi-trailers—and applications, multiple technologies offer opportunities to decarbonize MHDVs including battery-electric vehicles, hydrogen fuel cell vehicles, and sustainable liquid fuels. Here we provide an overview of the status, opportunities, challenges, and uncertainties for these competing—and potentially complementary—technologies, including consideration of supporting infrastructure and prospects for future success. We find a bright outlook for zero-emission vehicles and discuss remaining barriers and uncertainties around fleet decisions and changes to vehicle operation, infrastructure, manufacturing, and future fuel and technology trends that can be informed through analysis.

## Graphical abstract

**Figure:**
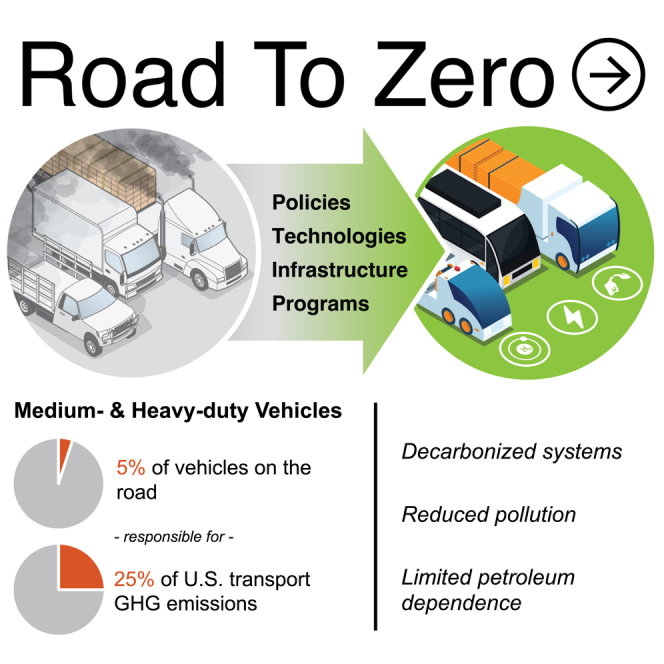

## Introduction

Despite representing only 5% of vehicles on the road, medium- and heavy-duty vehicles (MHDVs), with a gross vehicle weight rating above 10,000 pounds, account for nearly 30% of US on-road fuel consumption and emit almost 25% of total US transportation greenhouse gas (GHG) emissions.^1^^,^^2^ MHDVs include a large variety of vehicle types, ranging from heavy-duty pickup trucks and box trucks to full-size buses and Class 8 tractor semi-trailers, as detailed in Section 2. Virtually all MHDVs on the road in the US today use internal combustion engines, with over 80% fueled with diesel and the rest with gasoline.^1^ As a result, MHDVs are a major source of air pollutants that pose a significant health burden and disproportionally affect disadvantaged communities.^2^

Multiple technologies, at different stages of market readiness, offer opportunities to decarbonize MHDVs, including plug-in battery electric vehicles (BEVs), hydrogen fuel cell electric vehicles (FCEVs) using clean hydrogen, and internal combustion engine vehicles (ICEVs) using sustainable liquid fuels. Both BEVs and FCEVs are zero-emission vehicles (ZEVs), as they eliminate tailpipe emissions and associated air pollution.

BEVs, once considered impractical for MHDVs, have experienced significant cost reductions and technology improvements over the last decade, largely driven by reductions in battery costs.^3^^,^^4^ BEVs also offer performance advantages that can improve safety, reliability, and driver retention.^5^ The main drawbacks for medium- and heavy-duty BEVs remain their lower ranges, longer recharge times, and higher purchase prices compared to incumbent ICEVs.^6^^,^^7^ In addition, a lack of private and public charging infrastructure for MHDVs, lack of incentives to defray capital investments, and other fleet and manufacturing constraints are also responsible for the limited number of BEVs on the road today.^8^ However, many expect BEVs to become cost competitive for multiple MHDV applications in the near term, if not already, thanks to further reductions in battery costs combined with low operating costs from high power train efficiencies and reduced maintenance.^9^^,^^10^^,^^11^ BEVs are currently the most mature technology solution to decarbonize MHDVs, and many projections show BEV sales increasing rapidly over the next 10–15 years.^9^^,^^12^^,^^13^

Hydrogen FCEVs offer driving ranges and refueling times comparable to today’s diesel trucks. However, the costs to purchase these vehicles and produce clean hydrogen are currently much higher,^10^ and publicly accessible hydrogen refueling infrastructure remains effectively nonexistent in the US. Although these shortcomings may eventually be resolved, added energy conversion losses (specifically in the production of clean hydrogen and the in-vehicle conversion of hydrogen to electricity) give FCEVs an inherent disadvantage in “well-to-wheels” efficiency when compared to BEVs.^14^^,^^15^ Still, FCEVs can offer a compelling alternative to complement BEVs, especially for hard-to-electrify applications with challenging duty cycles like long-haul. FCEVs are less mature than today’s BEVs but could become a viable alternative if hydrogen technologies experience rapid improvements as batteries have. Ledna et al.^9^ suggest that if FCEV technologies progress in line with US Department of Energy (DOE) targets, a combination of BEVs and FCEVs could offer cost-competitive solutions for about 40% of MHDV sales in 2030 and a pathway to ZEV market dominance (i.e., 100% of sales) before 2050.

Finally, “drop-in” sustainable fuels such as advanced sustainable biofuels (liquid fuels produced in a sustainable manner from renewable biological sources, including wastes, plants, and algae) or e-fuels (liquid synthetic fuels produced using captured carbon and hydrogen produced by electrolysis of water with renewable electricity) can provide significant greenhouse gas emissions savings and do not require major changes to existing vehicles or fuel dispensing infrastructure. However, sustainable fuels will not reduce criteria air pollutant emissions (or resolve associated air quality concerns), and several constraints could limit their ability to scale up to fully replace petroleum in MHDV applications. Specifically, limited feedstock supply, unintended environmental consequences, and high costs could hinder the growth of sustainable biofuels, while significant electricity requirements, uncertain technology timelines, and high production costs could limit the role of e-fuels.^4^ Low-carbon fuels might eventually provide a solution for MHDV applications that prove especially challenging to decarbonize (through BEVs or FCEVs), as well as an opportunity to reduce emissions for legacy ICEVs as the transition to ZEVs unfolds.

This article offers a research and industry perspective on the status, opportunities, and challenges for various medium- and heavy-duty ZEV technologies in the US. We start by reviewing the current segmentation of the MHDV market (vehicle types and applications), including an overview of the current status and future outlook for ZEVs. Next, we review several key economic drivers and infrastructure barriers for transitioning to zero-emission MHDVs. We conclude by summarizing major takeaways and outlining several key uncertainties to be addressed in future analyses. Although the same technologies are used to decarbonize MHDV applications in other regions of the world, the freight and energy systems vary significantly across regions. Insights provided can inform other regions but might vary for places with different energy prices and availability, denser road networks that could more easily accommodate solutions like overhead catenary charging, or other differences that impact vehicle use, charging and fueling availability, and other key aspects required to transition to clean transportation solutions.

## MHDV market segmentation

**Figure 1:**
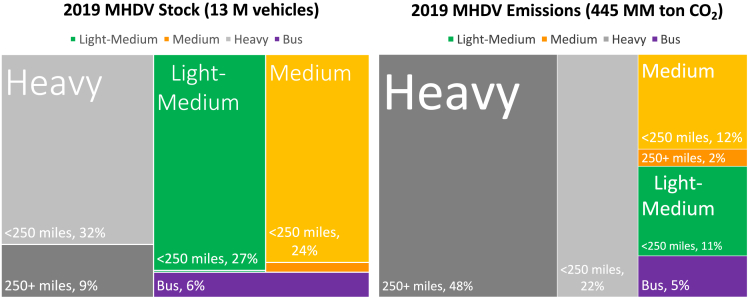
US medium- and heavy-duty vehicle stock (millions of vehicles on the road) and greenhouse gas emissions (millions of metric tons of CO_2_) by vehicle class and typical driving application The darker shades of color indicate long-haul operations (250+ miles), whereas lighter shades refer to local or regional operation. Source: Ledna et al.^9^

The US MHDV market is heterogeneous, reflecting a variety of needs and applications. Thus, multiple decarbonization solutions may be required to fully displace petroleum fuels. MHDV stock and emissions reflect the significant variation in vehicle characteristics and use across market segments. Vehicle stock (i.e., the number of vehicles on the road, left side of Figure 1) is divided roughly evenly across three MHDV segments, with light-medium trucks (Class 3) accounting for approximately 27%, medium trucks (Class 4–6) accounting for 25%, and heavy trucks (Class 7–8) accounting for 41%. Buses comprise the final 6%^16^ (passenger bus stock estimates are derived from passenger miles traveled and occupancy data^17^). Around 60% of trucks are used for freight movement (accounting for about 75% of total vehicle miles traveled [VMT]), with the rest used for other vocational applications. These applications are highly varied (*e.g.*, firefighting, tree trimming), each with their own unique driving and power requirements. The vast majority (approximately 90%) of MHDVs are used in applications with typical shipment distances (or driving distances for buses and vocational vehicles) of less than 250 miles,^9^^,^^18^^,^^19^ with about 10% of stock comprising heavy long-haul trucks with shipment distances greater than 250 miles.

Energy use and tailpipe emissions (right side of Figure 1) are not proportional to vehicle stock, given large variations in both vehicle fuel economy (large trucks consume more energy per mile) and vehicle use (i.e., annual VMT). For example, despite comprising only 9% of the vehicle stock, heavy long-haul trucks are responsible for an estimated 48% of tailpipe CO_2_ emissions, reflecting their lower fuel economies (per mile) and substantially higher VMT. Variations in vehicle stock, activity, and fuel economy suggest that different applications may require divergent technology solutions to decarbonize.

## Zero-emission MHDV market status and future outlook

The US market for medium- and heavy-duty ZEVs is at an early stage, trailing the light-duty market by several years. Most heavy-duty (Class 7–8) ZEV deployments in the US to date have been pilot demonstrations to familiarize fleet operators with the new technologies.^20^ A major exception is buses, with over 3,500 ZEVs in operation across the US—a 27% increase since 2020.^21^ Heavy-duty vehicles are considered the most challenging on-road vehicle segment to decarbonize for their heavy payloads, harsh duty cycles, high mileages, and mission constraints. However, recent studies show that certain applications, including short-haul delivery^22^ and drayage,^23^ can be economically electrified today through “short-range” BEVs (*i.e.*, ≤300-mile range) with depot-based charging at power levels commonly used for light-duty applications (*i.e.*, <150 kW). It is uncertain which ZEV technology will be most widely adopted for long-haul applications (9% of vehicles but 48% of energy use and emissions). Although BEVs carry an advantage over FCEVs in terms of overall efficiency and technology readiness, recent studies show that further improvements to battery range, charging speeds, and gravimetric energy densities will be needed to enable long-haul BEVs to operate similarly to diesel trucks today.^24^^,^^25^^,^^26^ Use of BEVs for long-haul operations is likely to require charging at the megawatt-level scale, and significant infrastructure investments, both in terms of cost, deployment time, and grid implications. The Charging Interface Initiative (CharIN) is expected to release the Megawatt Charging System standard in 2024 to create a common solution for high-power charging of commercial vehicles.^27^ Conversely, for FCEVs, long-haul operations are likely to be a key market application, but the cost and durability of fuel cells and the cost to produce zero-carbon hydrogen must improve. Both technologies will require large investments in charging/fueling infrastructure to enable adoption, especially for vehicles and fleets without access to private charging/fueling.

Despite today’s low adoption levels, traditional truck manufacturers and new technology providers are increasingly announcing plans to bring new ZEVs to market, whereas customers continue to lock in large preorders years before their expected delivery dates.^28^^,^^29^^,^^30^^,^^31^ Main drivers of early ZEV deployments include steady technology improvements, the availability of purchase incentives for vehicles (e.g., California Air Resources Board’s Hybrid and Zero-Emission Truck and Bus Voucher Incentive Project^32^ programs like Zero- and Near Zero-Emission Freight Facilities,^33^ the Inflation Reduction Act) and infrastructure (*e.g.*, California Energy Commission’s Energy Infrastructure Incentives for Zero-Emission Commercial Vehicles^34^ and utility make-ready programs), market development policies (*e.g.*, Low Carbon Fuel Standard^35^), and corporate decarbonization pledges.

Many analysts now project significant growth in future medium- and heavy-duty ZEV adoption, though the extent and timing vary. For example, in the International Energy Agency’s recent “Announced Policies” scenario, 12% of heavy-duty truck sales, 37% of medium-duty van sales, and 43% of bus sales are projected to be electric in the US by 2030.^1^ In their “Economic Transition Scenario,” BloombergNEF^7^ projects that BEVs and FCEVs will make up approximately 30% and 2% of global MHDV sales, respectively, by 2040. This includes nearly 80% BEV bus sales by 2040, representing an even more rapid transition than passenger vehicles. Ledna et al.^9^ show that with continued improvement in ZEV technologies, total-cost-of-driving parity with conventional vehicles is achievable by 2035 for all MHDV segments, and the trade-offs between BEVs and FCEVs largely depend on technology, performance, and fuel costs.

Excluding buses, three-fourths of zero-emission MHDVs deployed in the US from 2010 to 2021 were medium-duty BEVs.^20^ Medium-duty ZEV adoption is projected to lead heavy-duty adoption by several years thanks to lower range requirements requiring smaller battery sizes and reduced incremental BEV costs compared to ICEVs. In addition, the typical return-to-base operations of medium-duty vehicles provide easier access to low-cost private charging. Considering these operating characteristics and projected technology improvements, Ledna et al.^9^ find that electric vehicles are likely to become cost competitive for nearly all medium-duty vehicle applications before 2030.

## Economic drivers of zero-emission MHDV adoption

Although many factors influence technology adoption, economics will drive ZEV adoption for commercial applications to a greater extent than personal vehicles. Multiple studies use total cost of driving or ownership to project ZEV adoption, considering factors such as the upfront vehicle purchase price, fuel and maintenance costs, vehicle lifetime and discount rates, vehicle use, and occasionally additional factors like infrastructure availability and penalties for reduced payload capacity or increased refueling times (for BEVs in particular). Many of these assumptions are highly uncertain, including future technology cost and performance projections and the financial considerations that drive fleet decision-making. For example, the financial horizon over which fleet operators make their purchase decisions heavily impacts comparative cost calculations. Cost savings for ZEVs increase over time as the vehicles are driven more and operating costs become a more dominant factor in the total cost of ownership. That said, adoption models sometimes take a first-owner perspective and only consider the first few years of a vehicle’s life, which could under-represent the value of ZEVs over their entire life. In addition, assumed range requirements are a significant driver of modeled ZEV purchase prices, but these requirements (and their value) for individual fleets are uncertain and could change over time. The lower operating costs of ZEVs could induce changes to fleet logistics and vehicle use that are challenging to predict but might lead to reduced vehicle and operational costs.

**Figure 2:**
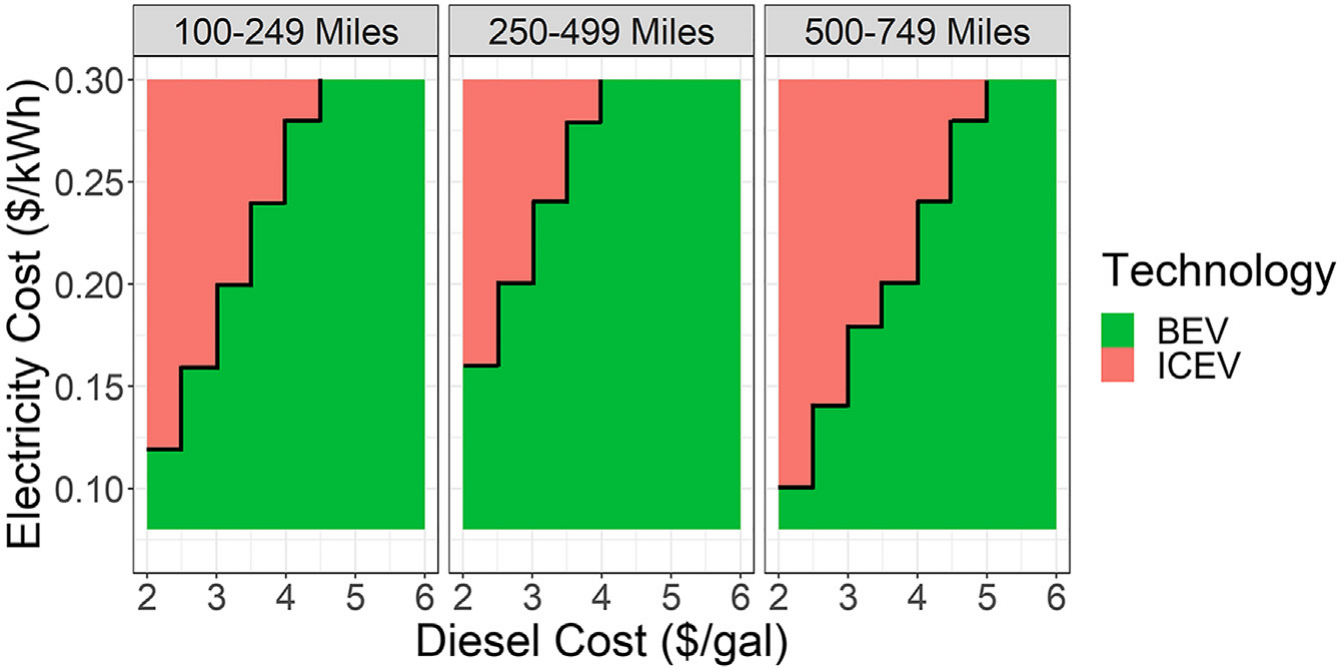
Least-cost technology between battery-electric and diesel heavy-duty trucks in 2035 as a function of diesel and electricity prices for different use cases (vehicle shipment distance bin), illustrating the complex dynamics between vehicle use and fuel prices (other technologies and fuels excluded for simplicity of visualization) Moving from the 100–249-mile bin to the 250–499-mile bin reflects higher VMT and greater operational cost savings for BEVs, leading to greater cost competitiveness. Applications in the 500–749-mile shipment distance bin require longer vehicle range (more expensive BEVs) and additional charging dwell times (monetized at $75/h here), which exceed savings from higher VMT and increase ICEV competitiveness.

Fuel costs are a key determinant of operating costs yet are also highly uncertain. Fossil fuel prices are highly volatile because of complex global geopolitical and market forces. There is also uncertainty around the price of clean hydrogen, given the present lack of markets and infrastructure. While less volatile, electricity prices will be influenced by location and time of day, affecting the cost of charging (and potential grid upgrades).^22^^,^^36^ Stationary energy storage and flexible BEV charging offer opportunities to reduce charging costs, but challenges remain to fully implement effective BEV-grid integration strategies at scale.^37^^,^^38^ Figure 2 illustrates the cost competitiveness of heavy-duty BEVs and ICEVs (including conventional diesel vehicles and hybrid electric vehicles) as a function of electricity and diesel prices based on 2035 technology and financial assumptions from the central scenario in Ledna et al.^9^ Trade-offs between fuel cost and least-cost technology vary by typical shipment distance, which incorporates differences in annual VMT and monetized dwell times for BEV charging. At higher diesel prices (greater than $4–$5/gallon), BEVs are economically advantageous to ICEVs when charging prices are between $0.10/kWh and $0.30/kWh. ICEVs remain more cost-effective when diesel prices are below $3/gallon for the majority of electricity prices considered. As driving distances increase, the opportunity cost of BEV charging becomes a more substantial factor, outweighing operational cost savings and reducing BEV competitiveness (this scenario monetizes the time spent charging at $75/h and assumes BEV charging at 500 kW, though higher charging speeds could reduce BEV dwell time and thus total cost).

## Infrastructure: Critical to enabling and scaling ZEV adoption

A major barrier to large-scale ZEV adoption today is the lack of fueling solutions and networks. ZEVs will not be adopted at scale if they cannot be reliably fueled, and building the required infrastructure will take multiple years and likely decades to achieve ubiquitous convenient refueling access for all vehicles and applications. Despite sharing some early market similarities, the refueling infrastructure to support BEVs and FCEVs will look quite different.

**Figure 3:**
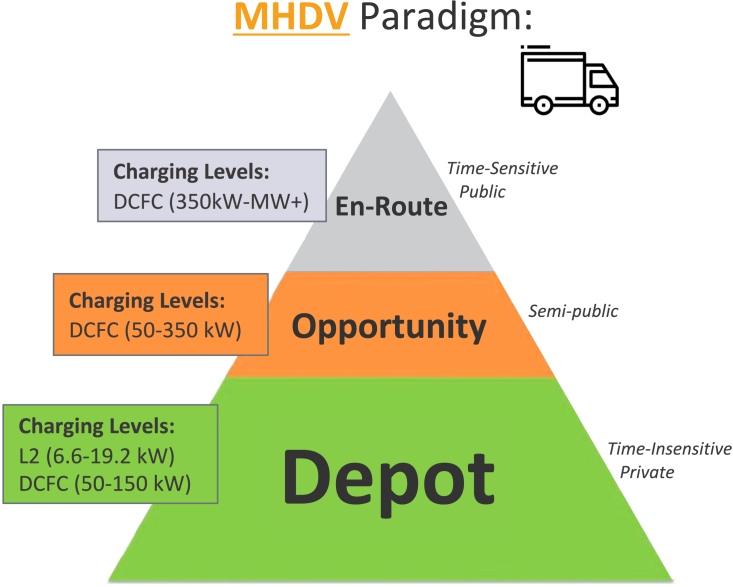
Battery-electric MHDV charging will be heterogeneous, consisting of low-power private depot, semipublic opportunity (e.g., during loading/unloading), and high-power public en route charging, depending on availability and application (DCFC: direct-current fast charging; L2: Level 2 charging) Source: Muratori and Borlaug,^39^ inspired from National Research Council.^40^

BEV charging for MHDVs is expected to be heterogeneous, matching specific operating behaviors and charging needs with a combination of slow private or semiprivate charging and fast public charging. BEVs can take advantage of extended downtimes (*e.g.,* loading/unloading) to charge at moderate power levels, usually referred to as “opportunity charging.” This could take place at public or semipublic locations where vehicles routinely dwell. For certain applications, BEVs may be able to perform all their charging while parked at their depot, with privately installed kilowatt-level charging equipment and sufficient temporal flexibility to further reduce electricity costs through managed charging.^22^ This is especially true for medium-duty vehicles such as delivery vans and buses, which have limited daily mileage requirements and consistent operating schedules, but also for heavy trucks used in short-haul applications (around 90% of MHDVs have typical shipment distances less than 250 miles, yet they only comprise about 50% of total MHDV energy, per Figure 1). Borlaug et al.^24^ show that even heavy-duty freight trucks are off-shift and potentially available to depot or opportunity charge for an average of 14–17 h per day. On the other hand, there will be a need for “en route fast charging,” similar to existing liquid (and potential future hydrogen) fueling stations, where a vehicle’s shift is interrupted to charge as quickly as possible (multiple hundreds of kilowatts to megawatt-level charging). This can be used to fill in the gaps for applications with particularly high mileage requirements (e.g., long-haul) or for BEVs without other charging options. Figure 3 depicts a potential charging paradigm for commercial medium- and heavy-duty BEVs.

Hydrogen refueling infrastructure, on the other hand, will largely resemble existing fossil fuel stations, with the main differences being that the hydrogen may be produced on-site and that hydrogen stations will have much higher electricity demands than existing diesel stations (for hydrogen compression and dispensing, and especially if hydrogen is produced on-site via electrolysis). A key barrier to zero-carbon FCEVs is the cost to produce clean hydrogen, currently about an order of magnitude higher than the $0.50–$1.70/kg for producing hydrogen from natural gas, by far the most popular method today.^41^ DOE has set a target for producing clean hydrogen at $1/kg by 2030 (for large-scale plants, excluding transport and dispensing), which would enable FCEVs to be cost competitive with ICEVs and BEVs.^42^ This would require major cost reductions for electrolyzers with widespread access to low-cost carbon-free electricity. If electrolysis isn’t performed on-site, the transmission and delivery of hydrogen poses another challenge for both cost and infrastructure deployment. Unlike BEV charging, which can largely leverage and expand the existing electricity grid, hydrogen stations must develop new zero-carbon supply chains for producing, transporting, and distributing hydrogen at scale. Currently, there are fewer than 50 hydrogen refueling stations in the US, with all but one located in California,^43^ and these typically rely on hydrogen deliveries from large central production plants. At today’s low volumes, delivery and dispensing of hydrogen is achieved using heavy-duty trucks, adding about $8–$11/kg to the hydrogen production cost,^44^ though DOE has set a target to reach $2/kg for hydrogen distribution and dispensing once large-scale production volumes have been achieved.^45^ Given low volumes, the final cost of hydrogen exceeded $15/kg at retail stations in 2022, even though the number of reporting stations was very limited.^46^

Both BEVs and FCEVs face a “chicken-and-egg” problem with respect to the deployment of charging/fueling infrastructure, but with electric truck production ramping up it is becoming more pressing to ensure that the required charging infrastructure is timely deployed and operated reliably to enable adoption and use of ZEVs. For example, the Port of Long Beach estimates that about 60% of drayage trucks could require public charging.^47^ However, today there are just a handful of publicly accessible sites for MHD charging across the nation. A key question that applies to both technologies is whether the requisite infrastructure will be in place by the time each technology’s total-cost-of-ownership tipping points are achieved and mass market demand is realized. BEVs have an early advantage for applications that do not require public fast charging and operate out of a private depot already connected to the electricity grid (though depot charging installations may still require costly and time-consuming upgrades to electricity distribution systems^22^). Behind-the-fence (i.e., private) chargers will be critical for the early adoption of BEVs in the MHDV segment, and financial support in the form of state agency grants, utility make-ready programs, BEV-friendly electricity tariffs, and rebates for charging infrastructure can all help to promote electrification. Technical standards for infrastructure performance and interoperability, as well as requirements for reliability and uptime, will be critical to instill consumer confidence in ZEVs. Finally, coordination between local governments and utilities to streamline and shorten the prolonged (several months to more than a year) permitting and interconnection processes for new ZEV infrastructure will be important to avoid market stagnation.^48^

## Conclusions

Decarbonizing MHDVs is a critical step for achieving climate and air quality goals and will require a rapid transition to ZEVs—possibly complemented by sustainable liquid fuels for certain applications and legacy vehicles. Effective collaboration across multiple stakeholders in the public and private space is critical to enable a full transition to ZEVs, supported by a multi-pronged approach that includes: (1) R&D investments to further improve technologies; (2) investments in manufacturing, supply chains, and supporting infrastructure; and (3) policies and regulation aimed at supporting clean technologies and reducing GHG and pollutant emissions.^49^ Despite limited market adoption to date, BEVs are commercially available and becoming an increasingly viable option for many MHDV applications, with multiple outlooks projecting widespread near-term adoption in the US. Main BEV advantages include superior performance that can improve safety and is compelling for drivers; high efficiency and reliability, reducing driving and maintenance costs; and zero-emission operations. FCEVs and sustainable fuels are at an earlier stage of market readiness but may become a viable solution for long-haul and other challenging-to-electrify applications, especially as major investments are being made to further develop these technologies and reduce costs. Policy support mechanisms, such as rebates and incentives, can help to jump-start ZEV adoption and deploy the required infrastructure by offsetting capital costs for the early adopters. For example, the landmark programs within the Infrastructure and Investment Jobs Act and Inflation Reduction Act will spur never-before seen ZEV activity in the US.^50^ In addition, many states, led by California, are growing and rolling out new incentive programs to foster the adoption of ZEVs. Charging and refueling networks must be expanded significantly, and system-level solutions will be needed to ensure interoperability and effective integration with electricity systems. Policies and regulations aimed at reducing GHG and pollutant emissions will help support a sustained market adoption provided they also address technical and implementation barriers such as accelerated permitting and utility interconnection for rapid ZEV infrastructure build out, charger reliability and interoperability, supply chain resiliency, and workforce development. Moreover, coordination between disparate and historically unconnected stakeholders, including state agencies, local governments, automotive manufacturers, fleets, energy infrastructure and utility companies, and research and academia will be required to ensure a smooth and timely transition to ZEVs. This paper, a joint research and industry perspective, is one such example of cross-sectoral collaboration.

Although the outlook for commercial medium- and heavy-duty ZEVs is bright, and ZEV technologies are steadily improving, several barriers and uncertainties remain to be explored.

• First, the trade-offs between various technology solutions within different applications remains unclear and will be largely dependent on uncertain technology and fuel cost trajectories and ever-changing fleet logistics and vehicle operations. This uncertainty can slow down investments and adoption of clean technologies. Further research into the decision-making criteria for various fleet operators could help to better understand and match fleet priorities with best-suited technologies and accelerate investments
• Second, significant infrastructure investments will be required to provide charging/refueling and to transport and deliver clean fuels to stations. Without reforms, the lack of streamlined and harmonized permitting processes, and very long lead times for energy infrastructure build out (up to several years) will complicate and delay the deployment of ZEV infrastructure, thereby slowing down the ZEV market transition. Moreover, the ability to scale up manufacturing and establish reliable supply chains for raw materials and critical rare-earth metals will require major investments^51^
• Third, although MHDVs can leverage economies of scale from other technologies sharing similar components and materials (e.g., light-duty ZEVs, stationary energy storage systems, and production of bioenergy and hydrogen for use in other sectors), competing demand for limited materials could lead to shortages that delay and/or increase the short-term costs for ZEVs. Research into solutions that enable the development of robust supply chains for minerals and materials critical to ZEVs, including end-of-life recycling processes, is highly warranted
• Fourth, large increases in clean electricity demand for BEVs, as well as hydrogen and e-fuel production, could prove challenging for the power system and will require careful planning. For biofuels, effective and fully sustainable production pathways will need to be demonstrated at scale

## Limitations of the study

Finally, this paper summarizes our perspectives on the current status and expectations for transitioning to zero-emission commercial vehicles, but difficult-to-foresee trends and disruptions could impact future mobility needs and the potential for various decarbonization technologies. For example, automation could enable increased vehicle utilization and shifting economics in favor of BEVs for their lower operating costs while also prioritizing faster recharge speeds to maximize asset utilization. The introduction of new advanced technologies and trends could disrupt freight demands, logistics, and vehicle usage patterns for MHDVs in a way that fundamentally reshapes the outlook for different decarbonization solutions. In general, zero-emission technologies use for commercial vehicles is still in its infancy, and data-driven analyses like the ones used to inform our perspectives will need to be updated to provide robust and timely insights to inform decision-making, especially in areas of high uncertainty.
